# Associations between *APOE* and low-density lipoprotein cholesterol genotypes and cognitive and physical capability: the HALCyon programme

**DOI:** 10.1007/s11357-014-9673-9

**Published:** 2014-07-30

**Authors:** Tamuno Alfred, Yoav Ben-Shlomo, Rachel Cooper, Rebecca Hardy, Cyrus Cooper, Ian J. Deary, Jane Elliott, David Gunnell, Sarah E. Harris, Mika Kivimaki, Meena Kumari, Richard M Martin, Chris Power, Avan Aihie Sayer, John M. Starr, Diana Kuh, Ian NM Day, the HALCyon Study Team

**Affiliations:** 1 School of Social and Community Medicine, University of Bristol, Canynge Hall, 39 Whatley Road, Bristol, BS8 2PS UK; 2 MRC Unit for Lifelong Health and Ageing, University College London, London, WC1B 5JU UK; 3 MRC Lifecourse Epidemiology Unit, University of Southampton, Southampton, SO16 6YD UK; 4 Institute of Musculoskeletal Sciences, University of Oxford, Oxford, OX3 7LD UK; 5 Centre for Cognitive Ageing and Cognitive Epidemiology, University of Edinburgh, Edinburgh, EH8 9JZ UK; 6 Department of Psychology, University of Edinburgh, Edinburgh, EH8 9JZ UK; 7 Centre for Longitudinal Studies, Department of Quantitative Social Sciences, Institute of Education, 20 Bedford Way, London, WC1H 0AL UK; 8 Medical Genetics Section, Molecular Medicine Centre, Institute of Genetics and Molecular Medicine, Western General Hospital, Crew Road, Edinburgh, EH4 2XU UK; 9 Department of Epidemiology and Public Health, University College London, London, WC1E 6BT UK; 10 MRC Centre for Causal Analyses in Translational Epidemiology, University of Bristol, Bristol, BS8 2BN UK; 11 Centre for Paediatric Epidemiology and Biostatistics, UCL Institute of Child Health, 30 Guilford Street, London, WC1N 1EH UK; 12 Academic Geriatric Medicine, Southampton General Hospital, University of Southampton, Southampton, SO16 6YD UK; 13 Alzheimer Scotland Dementia Research Centre, University of Edinburgh, 7 George Square, Edinburgh, EH8 9JZ UK

**Keywords:** Ageing, Apolipoprotein E, Cognition, Single nucleotide polymorphism

## Abstract

The *APOE* ε2/3/4 genotype has been associated with low-density lipoprotein cholesterol (LDL-C) and Alzheimer disease. However, evidence for associations with measures of cognitive performance in adults without dementia has been mixed, as it is for physical performance. Associations may also be evident in other genotypes implicated in LDL-C levels. As part of the Healthy Ageing across the Life Course (HALCyon) collaborative research programme, genotypic information was obtained for *APOE* ε2/3/4, rs515135 (*APOB*), rs2228671 (*LDLR*) and rs629301 (*SORT1*) from eight cohorts of adults aged between 44 and 90 + years. We investigated associations with four measures of cognitive (word recall, phonemic fluency, semantic fluency and search speed) and physical capability (grip strength, get up and go/walk speed, timed chair rises and ability to balance) using meta-analyses. Overall, little evidence for associations between any of the genotypes and measures of cognitive capability was observed (e.g. pooled beta for *APOE* ε4 effect on semantic fluency *z* score = −0.02; 95 % CI = −0.05 to 0.02; *p* value = 0.3; *n* = 18,796). However, there was borderline evidence within studies that negative effects of *APOE* ε4 on nonverbal ability measures become more apparent with age. Few genotypic associations were observed with physical capability measures. The findings from our large investigation of middle-aged to older adults in the general population suggest that effects of *APOE* on cognitive capability are at most modest and are domain- and age-specific, while *APOE* has little influence on physical capability. In addition, other LDL-C-related genotypes have little impact on these traits.

**Electronic supplementary material:**

The online version of this article (doi:10.1007/s11357-014-9673-9) contains supplementary material, which is available to authorized users.

## Introduction

Higher levels of low-density lipoprotein cholesterol (LDL-C) have been associated with a number of age-related conditions, including increased risk of coronary heart disease (Wilson et al. 1998). Although modification by diet (Kelley et al. 2012) and pharmacological interventions (Law et al. 2003) is possible, LDL-C is partly heritable (Matteini et al. 2010), with several identified genetic variants contributing to its interindividual variability. Among these, the ε2, ε3 and ε4 alleles of the *APOE* gene are probably the most widely studied. Encoding apolipoprotein E and playing a key role in lipid metabolism (Mahley and Rall 2000), several studies have observed that carriers of the *APOE* ε2 and ε4 alleles display lower and higher, respectively, levels of LDL-C compared with the most common ε3/ε3 genotype (Bennet et al. 2007). In addition, it has been long established that carriers of the ε2 alleles are at lower risk, and those with the ε4 alleles are at higher risk of Alzheimer disease (Bertram et al. 2007). Among cognitively healthy individuals, there is also evidence from many, though not all studies, that carriers of the ε4 allele perform worse in some cognitive tests and that these effects may be more pronounced in older populations (Caselli et al. 2009; Wisdom et al. 2011; Davies et al. 2014). For instance, there is little evidence to support the influence of the *APOE* genotype on cognitive measures in children and young people (Taylor et al. 2011; Ihle et al. 2012), though there is some mixed evidence for its influence in middle-aged (Zhao et al. 2005; Bunce et al. 2011) and older (Deary et al. 2004a) adults. In addition, there is some evidence from longitudinal studies that ε4 carriers experience greater cognitive decline in mid- (Blair et al. 2005) and later (Schiepers et al. 2012) life. *APOE* may also have a role in ageing phenotypes more generally, with other associations including triglycerides, apolipoproteins, C-reactive protein (Khan et al. 2013), type 2 diabetes (Anthopoulos et al. 2010), longevity (McKay et al. 2011; Nebel et al. 2011) and Parkinson disease (Lill and Roehr).

Given that the observed associations between cognitive and physical performance measures (Deary et al. 2006; Clouston et al. 2013) may be a result of genetic influences on shared age-related pathways simultaneously leading to the impairment of both (Johnson et al. 2009), it has been hypothesised that the pleiotropic *APOE* genotype may also affect physical performance. However, to date, investigations into associations between *APOE* and physical performance have also been inconsistent, with the ε4 allele reported to be associated with slower gait speeds (Melzer et al. 2005), but not with fitness (Deary et al. 2006), frailty (Rockwood et al. 2008), or grip strength (Vasunilashorn et al. 2013) and mixed findings for disability (Hyman et al. 1996; Kulminski et al. 2008) and chair stands speed (Melzer et al. 2005; Vasunilashorn et al. 2013) in middle-aged and older adults.

Therefore, further investigations into associations between *APOE* and cognitive and physical performance measures are required. It is also of interest to examine whether any such effects are related to an effect on LDL-C. Given the conflicting evidence for associations between levels of LDL-C and cognitive (Yaffe et al. 2002; Packard et al. 2007) and physical performance measures (Walston et al. 2002; Blain et al. 2012), investigations of other LDL-C-related genotypes may help to elucidate whether or not any effects are unique to *APOE*. Therefore, we analysed data from 23,916 participants aged between 44 and 90+ from eight UK cohorts as part of the HALCyon research programme (Healthy Ageing across the Life Course; www.halcyon.ac.uk) to investigate associations between the *APOE* genotype and objective measures of physical and cognitive capability, the capacity to undertake the physical and mental tasks of daily living. We also explored whether any effects are also demonstrated for other genotypes robustly associated with LDL-C (rs515135 (*APOB*), rs2228671 (*LDLR*) and rs629301 (*SORT1*)) in genome-wide association studies (Aulchenko et al. 2009; Teslovich et al. 2010) (GWAS).

## Methods

### Study populations

Participants from eight UK cohorts of the HALCyon programme were included in this report. The National Child Development Study (NCDS) has followed up all individuals born in England, Scotland and Wales during 1 week in March 1958. In 2002–2004, a Biomedical Survey was conducted during home visits by a research nurse. Following informed consent, DNA was extracted from 8,017 participants aged 44–45 years; the sample with immortalised cell line culture (*n* = 7,526) is used here. In 2008-2009, an eighth sweep was carried out during which cognitive performance tests were conducted (Brown and Dodgeon 2010). Further details of the study are available elsewhere (Power and Elliott 2006).

The Medical Research Council National Survey of Health and Development (NSHD) comprises participants sampled from all births in a week in March 1946 and followed up since. In 1999, at age 53 years, men and women were visited by a research nurse and DNA was extracted from 2,756 members of the cohort. Details of the data collected and the several phases of the study are available on the cohort’s website (www.nshd.mrc.ac.uk) and elsewhere (Wadsworth et al. 2006).

The Whitehall II study targeted all civil servants aged between 35 and 55 years working in London in 1985–1988. In 2002–2004 (phase 7), the genetics study was established and DNA was extracted from 6,156 participants. Details of the study design and data collected have been described elsewhere (Marmot and Brunner 2005).

The Caerphilly Prospective Study (CaPS) recruited 2,512 men aged between 45 and 59 years in 1979–1983 from the town of Caerphilly, South Wales and its surrounding villages. Blood samples were collected at baseline and at each of the four follow-ups (phase II, 1984–1988; phase III, 1989–1993; phase IV, 1993–1997 and phase V, 2002–2004). Further details are available on the cohort’s website (http://www.bris.ac.uk/social-community-medicine/projects/caerphilly).

The English Longitudinal Study of Ageing (ELSA) comprises men and women aged 50 years and over who originally participated in the Health Survey for England in 1998, 1999 or 2001. Fieldwork began in 2002–2003 (phase I) with two yearly follow-ups, and in 2004–2005 (phase II), blood samples were provided by 6,231 participants. Details of the cohort have been published elsewhere (Marmot et al. 2003).

The Hertfordshire Cohort Study (HCS) consists of 2,997 participants born 1931–1939 and registered with a general practitioner in East, North or West Hertfordshire who attended a clinic in 1994–2004 (phase I). A second assessment took place in 2004–2005 for participants in East Hertfordshire (phase II). Further details of the study design, data collected and summaries of participant characteristics have been published (Syddall et al. 2005) and are available on its website (www.mrc.soton.ac.uk/herts).

The Boyd Orr cohort is a historical cohort study based on children surveyed in 1937–1939 in English and Scottish districts. Participants were followed up for vital status via the NHS Medical Information Research Service (MIRS) since 1948, with questionnaire administration to survivors in 1997–1998 (phase II) and a research clinic visit in 2002–2003 (phase III), during which DNA was extracted from 728 adults, of which 397 of 405 who attended specific measurement research clinics had at least one physical capability measure and one relevant genotype called for this analysis. Details of the study design and the data collected have been described on its website (http://www.bris.ac.uk/social-community-medicine/projects/boyd-orr) and elsewhere (Martin et al. 2005).

The Lothian Birth Cohort 1921 (LBC1921) participants were all born in 1921 and most completed a cognitive ability assessment at age 11 years. In 1999–2001 (wave I), at approximately 79 years old, 550 participants living in and around Edinburgh, underwent a series of cognitive and physical tests. In 2003–2005 (wave II), 321 returned at 83 years old. Details of the recruitment into the study are available on its website (www.lothianbirthcohort.ed.ac.uk) and have been published previously (Deary et al. 2004b; Deary et al. 2012).

### Genotyping and quality control

Single nucleotide polymorphisms (SNPs) rs7412 and rs429358 form the *APOE* genotype (ε2/ε2, ε2/ε3, ε2/ε4, ε3/ε3, ε3/ε4 and ε4/ε4; 19q13.32) and genotyping was carried out by KBioscience (www.lgcgenomics.com) in Boyd Orr, CaPS, HCS, NCDS and NSHD. In ELSA, two TaqMan assays (rs429358 and rs7412, Assay-On-Demand, Applied Biosystems, Geneservice Ltd, Cambridge, UK) were used and run on a 7900HT analyzer (Applied Biosystems), and genotypes indicated by the Sequence Detection Software version 2.0 (Applied Biosystems). Details have been described previously in LBC1921 (Schiepers et al. 2012) and Whitehall II (Sabia et al. 2010). Genotyping for other SNPs (rs515135 (*APOB*, 2p24.1), rs2228671 (*LDLR*, 19p13.2) and rs629301 (*SORT1*, 1p13.3)) in ELSA, HCS, CaPS and Boyd Orr were carried out by KBioscience, as well as for rs2228671 in NSHD. In LBC1921, information came from the Illumina Human 610-Quadv1 Chip (using proxies rs541041 for rs515135 and rs646776 for rs629301) (Houlihan et al. 2010). SNPs in NCDS came from two sources—Illumina HumanHap 550 k v3 and Illumina 1.2 M chips (using rs562338 and rs541041 as proxies for rs515135 and rs646776 as a proxy for rs629301) (Wellcome Trust Case Control Consortium 2007). Genotype information for these three SNPs in Whitehall II as well as rs515135 and rs2228671 in NSHD came from the Illumina Metabochip (www.illumina.com). Genotypic data quality was reviewed by assessing departure from the Hardy-Weinberg equilibrium (HWE), clustering quality (using KBioscience software SNPviewer on their data) and call rates.

### Phenotypes

#### Cognitive capability

A number of cognitive performance tests in the different studies were used to assess cognitive capability. Different assessments of verbal memory (Bopp and Verhaeghen 2005) were conducted: in ELSA and NCDS, a list of 10 common words were used, with participants asked to recall the list immediately and again after a delay of around 5 min; the mean score was used in the analysis; in NSHD, 15 words were used over three trials; in Whitehall II, 20 words were used; responses in NSHD and Whitehall II were given in writing. In Whitehall II, participants recalled in writing in 1 min as many words as possible beginning with ‘S’ to assess phonemic fluency (Borkowski et al. 1967), while in LBC1921 three letters ‘C’, ‘F’ and ‘L’ were used with responses given orally. Participants were asked to recall as many animals as possible within 1 min to measure semantic fluency (Borkowski et al. 1967); responses were given orally in ELSA, NCDS, CaPS and NSHD, and in writing in Whitehall II. To assess search speed (Richards et al. 1999), 1-min letter searches among grids of letters were used, 600 letters in NSHD and 780 in ELSA and NCDS.

#### Physical capability

Grip strength was measured in NSHD, ELSA, HCS and LBC1921 using electronic or hydraulic dynamometers, with the best measure used in the analysis where more than one trial was conducted. Standing balance tests were conducted in the studies, with participants’ eyes open: flamingo (up to a maximum time of 30 s) (Sport 1993), in NSHD, HCS, Boyd Orr and CaPS, and side-by-side, semi-tandem and full tandem (Stevens et al. 2008) in ELSA. Ability to balance was defined in this analysis as the ability to complete 30 s of the flamingo or 5 s of the tandem test. The timed get up and go test (Podsiadlo and Richardson 1991) was carried out in HCS, Boyd Orr and CaPS and required participants to get up from a chair, walk 3 m at a normal pace, turn, walk back, turn and sit down. Timed walks over 2.44 m (8 ft) at a normal pace and 6 m at a fast pace were carried out in ELSA and LBC1921, respectively. Speeds were calculated for timed walks and get up and go, with the fastest speeds used in the analysis where more than one trial was conducted. Timed chair rises (Csuka and McCarty 1985) involved asking participants to rise from a chair and sit back down 5 times in ELSA and HCS and 10 times in NSHD; the reciprocal of time taken in seconds × 100 (Kuh et al. 2005) was used in the analysis. Further details of these measurements in these cohorts are presented elsewhere (Cooper et al. 2011).

### Statistical methods

Where information on ethnicity was collected, non-European participants were excluded from analyses in order to avoid confounding from population stratification (Cordell and Clayton 2005). Within studies, linear regression analyses were conducted on the continuous traits and logistic regression analyses were conducted on the ability to balance, adjusting for age, except in the age homogeneous cohorts of NCDS, NSHD and LBC1921 as well as sex in all cohorts except CaPS. *APOE* genotype was analysed here in two ways: first, ε2+ (ε2/ε2 or ε2/ε3) and ε4+ (ε3/ε4 or ε4/ε4) were compared with ε3/ε3; second, ε4+ (ε3/ε4 and ε4/ε4) was compared with non ε4 (ε2/ε2, ε2/ε3 and ε3/ε3). Due to the differences observed on LDL-C for carriers of the ε2 and ε4 alleles (Bennet et al. 2007), the ε2/ε4 genotype was excluded from the *APOE* analysis. Additive models were used for SNPs rs515135 (*APOB*), rs2228671 (*LDLR*) and rs629301 (*SORT1*), with genotypes coded as 0, 1 and 2 for the number of LDL-raising alleles. Likelihood ratio tests were used to compare the fit of the additive models compared with the full genotype model. Allelic scores were derived from counts of the number of *APOE* ε4 or LDL-C-raising alleles. For continuous traits, the normality of the standardised residuals was inspected with distributional diagnostic plots. For the harmonisation of continuous traits that were used to obtain pooled estimates of the genotypic effects, *z* score units were calculated in each study by subtracting the study mean and dividing by its standard deviation. The overall mean for *z* scores is 0 and standard deviation 1. Two-step (Riley et al. 2010) meta-analyses using a random-effects model were performed to obtain pooled genotypic effects. The *I* ^2^ measure was used to quantify heterogeneity (Higgins et al. 2003). Analyses of the interaction between ε4 carrier status and age within studies were also conducted. Where outcomes were collected at more than one phase, within-study analyses were performed on the annual change in scores using differences between the most recent and earliest measure available. Finally, the calculation of *z* scores, for the continuous traits, and the main analyses were repeated in men and women separately. Reporting of the analyses met the appropriate items of recommended checklists (Stroup et al. 2000; Little et al. 2009). A two-tailed significance level of *p* < 0.05 was used as evidence of statistical significance. Statistical analysis was performed in Stata 12.1 (StataCorp LP). Quanto (Gauderman and Morrison 2006) was used for power calculations.

## Results

### Cohort summaries and genotyping quality

A total of 23,916 adults aged between 44 and 90 + years had at least one relevant genotype and phenotype available (Table 1). Allelic frequencies were similar across the studies. Of the 23,061 participants with available *APOE* data, the genotypic frequencies were as follows: ε2/ε2—0.6 %, ε2/ε3—12.3 %, ε2/ε4—2.6 %, ε3/ε3—58.5 %, ε3/ε4—23.6 % and ε4/ε4—2.4 %. The HWE condition was met in all studies for all polymorphisms (*p* values > 0.1), except for rs515135 (*APOB*) and rs2228671 (*LDLR*) in NSHD and rs515135 (*APOB*) in ELSA (*p* values = 0.04). Summaries of measures of cognitive and physical capability are presented in Table S1.

**Table 1:** Summary of sex, age and LDL-related genotypes by cohort

	Cohort								
Characteristic	NCDS	NSHD	Whitehall II	CaPS	ELSA	HCS	Boyd Orr	LBC1921	Total
Number of participants	7,363	2,674	3,143	1,318	5,612	2,893	397	516	23,916
Male, %	50	50	77	100	46	53	45	41	55
Age^a^ in years, median (range)	44	53	59 (50–73)	61 (52–71)	65 (52–90+)	66 (59–73)	70 (64–82)	79 (77–80)	56 (44–90+)
*APOE* ε4 carrier status^b^, %	27	28	26	26	26	26	27	24	27
LDL-C-raising allele frequency									
rs515135 *APOB*, C	0.82	0.82	0.82	0.82	0.82	0.82	0.82	0.85	0.82
rs2228671 *LDLR*, C	0.87	0.88	0.87	0.88	0.88	0.88	0.88	0.88	0.87
rs629301 *SORT1*, A	0.78	0.77	0.79	0.77	0.78	0.78	0.77	0.75	0.78

Number of participants represents those with available data for at least one phenotype and at least one genotype

*CaPS* Caerphilly Prospective Study, *ELSA* English Longitudinal Study of Ageing, *HCS* Hertfordshire Cohort Study, *LBC1921* Lothian Birth Cohort 1921, *NCDS* National Child Development Study, *NSHD* National Survey of Health and Development

^a^Age at phase from which the majority of variables are taken, i.e. Boyd Orr: III; CaPS: III; ELSA: II; HAS: I; HCS: I; LBC1921: I; NCDS: Biomedical Survey (2002); NSHD: 1999 Collection; Whitehall II: VII

^b^ε4 carrier defined as ε3/ε4 or ε4/ε4; ε2/ε4 excluded from denominator

### Associations between *APOE* genotypes and cognitive capability

Figures S1–S4 present the associations between the cognitive capability measures and ε2+ and ε4+, adjusting for age and sex, using ε3/ε3 as the reference group in the cross-sectional analyses. There was no evidence for associations in the pooled analyses. Within studies, there was evidence for negative effects of ε2+ and ε4+ in NSHD and ELSA, respectively, on word recall (Figure S1), and of ε2+ in ELSA on semantic fluency (Figure S3). Table 2 shows that there was no evidence for associations between *APOE* and the cognitive capability measures when comparing carriers of the ε4 allele with noncarriers. Table S2 presents the findings of the interactions between the ε4 allele and age within the studies. In ELSA, there was some evidence that a negative effect of ε4 on word recall increases with age (*p* value = 0.04) and borderline evidence for this on search speed (*p* value = 0.08). There was some evidence that the effects of ε4 allele on word recall differs between men and women (*p* value for heterogeneity = 0.009; Figure S5). There was no evidence for heterogeneity between men and women for other outcomes (*p* values > 0.1; data not shown). For the few studies with data available, there was no strong evidence that the ε4 allele was associated with cognitive decline within the time periods investigated (Table S3).

**Table 2:** Summary of pooled associations between LDL-C-related genotypes and cognitive capability

Measure	Genotype	Beta (95 % CI)	*p*	*I* ^2^ %; Het *p*	*N*
	*APOE* ε4	−0.01 (−0.06–0.04)	0.67	46.0; 0.14	17,513
	rs515135 *APOB*	−0.02 (−0.04–0.01)	0.23	0.0; 0.67	16,473
Word recall	rs2228671 *LDLR*	0.01 (−0.02–0.04)	0.49	0.0; 0.97	16,481
	rs629301 *SORT1*	0.01 (−0.02–0.03)	0.52	0.0; 0.97	16,494
	*APOE* ε4	−0.01 (−0.16–0.14)	0.89	53.6; 0.14	3,528
Phonemic fluency	rs515135 *APOB*	0.00 (−0.07–0.07)	0.96	7.8; 0.30	3,638
	rs2228671 *LDLR*	−0.03 (−0.10–0.05)	0.52	6.8; 0.30	3,638
	rs629301 *SORT1*	−0.01 (−0.06–0.05)	0.83	0.0; 0.88	3,636
	*APOE* ε4	−0.02 (−0.05–0.02)	0.31	0.0; 0.98	18,796
Semantic fluency	rs515135 *APOB*	−0.02 (−0.05–0.01)	0.16	0.0; 0.97	17,761
	rs2228671 *LDLR*	−0.03 (−0.06–0.00)	0.048	0.0; 0.51	17,766
	rs629301 *SORT1*	0.01 (−0.04–0.05)	0.83	66.8; 0.02	17,774
	*APOE* ε4	0.00 (−0.05–0.06)	0.91	54.6; 0.11	14,348
Search speed	rs515135 *APOB*	−0.02 (−0.05–0.01)	0.26	0.0; 0.82	13,229
	rs2228671 *LDLR*	0.01 (−0.02–0.05)	0.54	0.0; 0.81	13,235
	rs629301 *SORT1*	0.00 (−0.05–0.05)	0.90	64.7; 0.06	13,250

Coefficients based on *z* scores and adjusted for age and sex. Coefficients for *APOE* ε4+: carrier vs. non-ε4 carrier; else per LDL-C-raising allele

### Associations between *APOB*, *LDLR* and *SORT1* genotypes and cognitive capability

There was some evidence that the LDL-C-raising allele of rs2228671 (*LDLR*) was associated with lower semantic fluency scores (Table 2, Figure S6). There was no evidence for any other associations between the SNPs and measures of cognitive capability (Table 2). Table S4 that shows there was no evidence for associations between any of the measures and the allelic count of the number of LDL-C-raising and ε4 alleles. There was no evidence for differences in genotypic effects by sex for the SNPs (*p* values > 0.06; data not shown).

### Associations between *APOE* genotypes and physical capability

Figures S7 to S10 show that there was no strong evidence of associations in the pooled analyses between the physical capability measures and ε2+ and ε4+ adjusting for age and sex, compared with ε3/ε3 in the cross-sectional analyses. Within studies, there was evidence in ELSA that participants with ε4+ had slower chair rise times than those with ε3/ε3 (Figure S9). There was no evidence that carriers of ε4 had poorer performance than noncarriers in the pooled analyses (Table 3). Table S5 shows that there was no evidence for interactions between the ε4 allele and age within the studies. There was no evidence for heterogeneity between men and women in the effects of the ε4 allele for these measures (*p* values > 0.6; data not shown). In the longitudinal analyses, there was evidence in ELSA that the ε4 allele was associated with decline in timed walk speed (*p* value = 0.02; Table S6).

**Table 3:** Summary of pooled associations between LDL-C-related genotypes and physical capability

Measure	Genotype	Beta (95 % CI)	*p*	*I* ^2^ %; Het *p*	*N*
	*APOE* ε4	0.01 (−0.02–0.05)	0.42	30.7; 0.23	10,646
Grip strength	rs515135 *APOB*	0.00 (−0.04–0.03)	0.81	41.5; 0.16	11,247
	rs2228671 *LDLR*	0.01 (−0.01–0.04)	0.40	0.0; 0.65	11,234
	rs629301 *SORT1*	−0.01 (−0.03–0.02)	0.61	34.1; 0.21	11,242
Get up and go/walk speed	*APOE* ε4	−0.02 (−0.07–0.04)	0.57	7.8; 0.36	6,810
	rs515135 *APOB*	0.00 (−0.05–0.06)	0.90	30.6; 0.22	7,291
	rs2228671 *LDLR*	0.04 (−0.06–0.13)	0.47	67.6; 0.01	7,277
	rs629301 *SORT1*	0.01 (−0.03–0.04)	0.73	0.0; 0.42	7,281
Timed chair rises	*APOE* ε4	−0.04 (−0.10–0.02)	0.18	37.8; 0.20	8,159
	rs515135 *APOB*	−0.03 (−0.07–0.01)	0.11	0.0; 0.85	8,601
	rs2228671 *LDLR*	0.00 (−0.05–0.04)	0.86	2.4; 0.36	8,598
	rs629301 *SORT1*	−0.02 (−0.06–0.01)	0.16	0.0; 0.47	8,601
	Genotype	OR (95 % CI)	*p*	*I* ^2^ %; Het *p*	*n*/*N*
Ability to balance ≥5 s	*APOE* ε4	1.02 (0.88–1.17)	0.80	0.0; 0.63	8,696/10,114
	rs515135 *APOB*	1.01 (0.91–1.13)	0.86	0.0; 0.67	9,131/10,654
	rs2228671 *LDLR*	1.04 (0.92–1.18)	0.54	0.0; 0.70	9,122/10,643
	rs629301 *SORT1*	0.97 (0.87–1.07)	0.49	0.0; 0.92	9,139/10,652

Coefficients for continuous outcomes based on *z* scores. Adjusted for age and sex. Coefficients for *APOE* ε4+: carrier vs. non-ε4 carrier; else per LDL-C-raising allele

### Associations between *APOB*, *LDLR* and *SORT1* genotypes and physical capability

Table 3 shows that there was no evidence for associations between the SNPs and measures of physical capability. Table S7 shows that there was evidence for associations between the allelic count of the number of LDL-C-raising and ε4 alleles and poorer timed chair rises (*p* value = 0.01). There was some evidence that the effect of the C allele of rs515135 (*APOB*) on timed chair rises differed by sex, with an inverse association observed in females only (*p* value for heterogeneity = 0.01; Figure S11). There was also evidence for heterogeneity by sex for the effects of rs2228671 (*LDLR*) on ability to balance (*p* value for heterogeneity = 0.04; Figure S12).

## Discussion

In this investigation of 23,916 adults aged between 44 and 90+ from eight UK cohorts, we examined the genotypic effects of the *APOE* ε2/3/4 genotype on measures of cognitive and physical capability, as well as those of three other LDL-C-related genotypes: rs515135 (*APOB*), rs2228671 (*LDLR*) and rs629301 (*SORT1*). For the four cognitive capability measures studied (word recall, phonemic fluency, semantic fluency and search speed), we found little evidence for associations with *APOE* genotype (Table 2). However, within-study investigations into interactions between ε4 and age provided suggestive evidence for an adverse effect of ε4 on word recall that increased with age (*p* value = 0.044, *n* = 4,971) as well as suggestive evidence for this on search speed in the largest age-heterogeneous cohort (*p* value = 0.08, *n* = 4,901; Table S2). In the longitudinal analyses, we observed no evidence that the ε4 allele was associated with cognitive decline. Investigations into the other genotypes revealed only borderline evidence for an association between the LDL-C-raising allele of rs2228671 (*LDLR*) and reduced semantic fluency (*p* value = 0.048, *n* = 17,766). There was little evidence to support associations between the *APOE* genotype and our physical capability measures (grip strength, timed get up and go/walk speed, timed chair rises and ability to balance) in the cross-sectional analysis and no evidence that genotypic effects vary by age. In the longitudinal analysis, there was evidence from ELSA that the ε4 allele was associated with decline in timed walk speeds (*p* value = 0.02, *n* = 2,195; Table S6). We found little evidence for associations between the other LDL-C-related genotypes and physical capability (Table 3).

Previous investigations into associations between the *APOE* genotype and measures of cognitive performance have produced mixed findings, possibly reflecting differences in cognitive domains and age (Wisdom et al. 2011). Meta-analyses of previous studies have found evidence for small adverse effects of the ε4 allele on some cognitive domains, including episodic memory, as well as evidence that this effect may increase with age (Wisdom et al. 2011). An increasing effect with age has also been seen in a study of over 10,000 Korean older adults (Shin et al. 2014). However, no evidence was found for effects on measures on verbal ability (Wisdom et al. 2011). Results from our investigations on *APOE* are consistent with many of those findings, with evidence for an interaction between ε4 and age on a measure of episodic memory and word recall and no evidence for any effects on our measures of verbal ability: semantic and phonemic fluency. Previous studies have observed a greater cognitive decline among carriers of the ε4 allele compared with noncarriers; in particular, there is evidence from a middle-aged cohort for differences in delayed word recall over 6 years (Blair et al. 2005), and a longitudinal study showed that declines in memory scores may be apparent before 60 years (Davies et al. 2014). Our own investigation from the model in ELSA suggested a detectable difference from around 55 years. Fewer investigations have been reported on the associations between *APOE* and measures of physical performance; however, findings include no association with athletic performance (Tsianos et al. 2010) and associations between ε4 and slower gait speeds from a study of 1,262 adults aged over 65 years (Melzer et al. 2005). Our larger investigation found evidence to support a role of *APOE* in decline in walk speeds over a 4-year period in one study but little evidence for associations with physical capability from cross-sectional analysis. Differences have been observed in the genotypic frequencies of rs11668477 in *LDLR* for mild cognitive impairment status in a small study of 114 cases and 92 controls (Liu et al. 2012). We believe that this is the largest investigation between *APOB*, *LDLR* and *SORT1* genotypes and measures of cognitive and physical performance.

Our large investigation into associations between the *APOE* genotype on cognitive capability measures provided only modest evidence for an adverse effect of the ε4 allele. There was also little evidence from the three other investigated LDL-C-related genotypes, suggesting that the small effects of *APOE* observed previously on some cognitive measures in population-based studies may not extend to genotypes implicated in LDL-C levels as a whole. However, the lack of evidence from our studies for these other SNPs may reflect a lack of power to observe possible small effects. Sample size calculations demonstrated around 11,000 individuals would be required to detect a beta coefficient of 0.05 *z* score units with 80 % power at the 5 % significance level for a SNP such as rs515135 (*APOB*) using an observed minor allele frequency of 0.18. We were sufficiently powered to detect differences as small as this for three of our cognitive measures; however, the observed pooled coefficients were much smaller. There are hypothesised mechanisms through which *APOE* may affect cognitive performance in non-demented adults other than through LDL-C, for instance, ε4/ε4 has been associated with increased rate of hippocampal volume loss (Crivello et al. 2010) and increased white matter hyperintensity burden (Schilling et al. 2013). Furthermore, *APOE* has been associated with several other ageing phenotypes (Khan et al. 2013). Our results suggest that these mechanisms might not lead to cognitive differences at a population level. Similar to the reports on cognitive performance (Yaffe et al. 2002; Packard et al. 2007), associations between measures of LDL-C and physical performance in the literature have been mixed, with higher levels associated with slower walking speeds (Walston et al. 2002), but some evidence that it may be protective against frailty (Blain et al. 2012). Our findings from other LDL-C-related genotypes suggest that LDL-C may not be an important mechanism through which *APOE* could affect physical performance. However, other potential pathways include the modest effects on specific cognitive measures and their decline and effects on bone-related phenotypes, though evidence for the latter is inconsistent (Peter et al. 2011; Tolonen et al. 2011).

Despite the size of our study, large age range and the inclusion of other LDL-C-related genotypes, there are some limitations. First, our ability to detect effects of our genotypes on decline in cognitive and physical capability may have been hindered by generally short intervals between measures in the cohorts (often around four years). Second, despite the important cognitive measures included here, there may be other cognitive domains which may be influenced by the *APOE* genotype. Third, although we only observed modest evidence for a detrimental impact of the ε4 allele on cognitive outcomes, it is possible that an effect may be influenced a likely higher proportion of preclinical dementia cases among the ε4 carriers. However, it is possible that *APOE* also affects non-pathological cognitive ageing (Davies et al. 2014). Fourth, we did not conduct investigations into the differential effects among each of the six *APOE* genotypes; however, given we observed only modest evidence for differences between ε4 and non-ε4 carriers, it is unlikely that this study would have been sufficiently powered to observe any of the putatively smaller differences. Furthermore, larger meta-analyses were unable to detect differences between the effects of ε3/ε4 and ε4/ε4 on several cognitive domains (Wisdom et al. 2011).

## Conclusion

This large, multi-cohort investigation into associations between *APOE* ε2/3/4 genotype, three other LDL-C-related SNPs and measures of cognitive and physical capability provided little support for anything but modest effects of these genotypes on these traits in middle-aged to older adults.

## Electronic supplementary material

Below is the link to the electronic supplementary material.

ESM 1 (DOC 29 kb)

ESM 2 (DOC 29 kb)

ESM 3 (DOC 42 kb)

ESM 4 (DOC 41 kb)

ESM 5 (DOC 32 kb)

ESM 6 (DOC 44 kb)

ESM 7 (DOC 42 kb)

ESM 8 (DOC 29 kb)

ESM 9 (DOC 42 kb)

ESM 10 (DOC 43 kb)

ESM 11 (DOC 47 kb)

ESM 12 (DOC 48 kb)

ESM 13 (DOC 40 kb)

ESM 14 (DOC 34 kb)

ESM 15 (DOC 48 kb)

ESM 16 (DOC 32 kb)

ESM 17 (DOC 39 kb)

ESM 18 (DOC 37 kb)

ESM 19 (DOC 34 kb)
