# Supplementary material for: Associations between APOE and low-density lipoprotein cholesterol genotypes and cognitive and physical capability: the HALCyon programme
Source: Age (Dordr). 2014 Jul 30;36(4):9673. doi: 10.1007/s11357-014-9673-9 (PMC4150901; doi:10.1007/s11357-014-9673-9)
Supplement: Supplementary file 2 — (DOC 29 kb) [file 11357_2014_9673_MOESM2_ESM.doc]

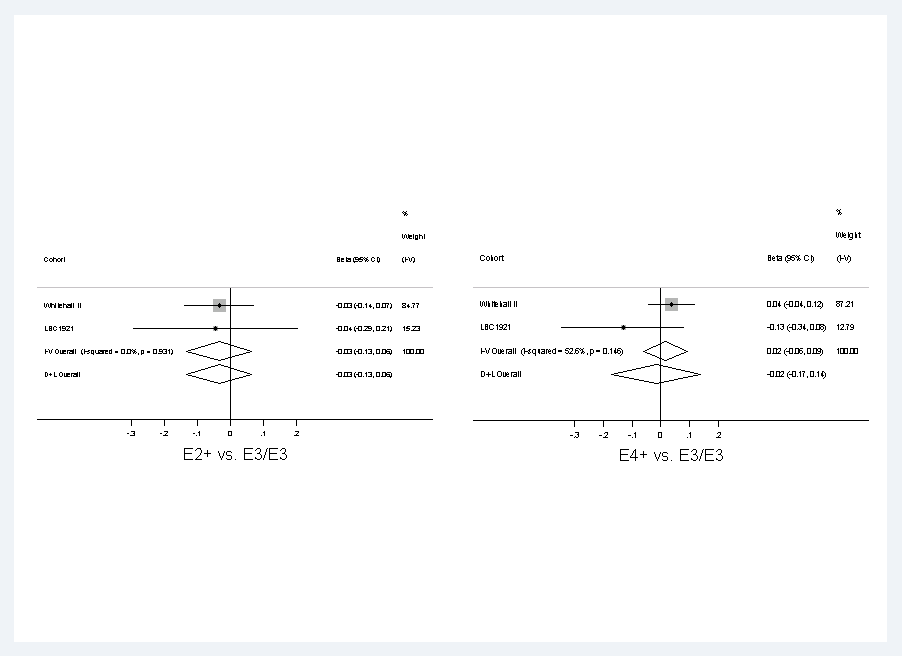


**Figure S2 Meta-analysis for the Association between *APOE* genotype and Phonemic Fluency**

Adjusted for age and sex. ε2/ε4 participants were excluded from this analysis
