# Supplementary material for: Associations between APOE and low-density lipoprotein cholesterol genotypes and cognitive and physical capability: the HALCyon programme
Source: Age (Dordr). 2014 Jul 30;36(4):9673. doi: 10.1007/s11357-014-9673-9 (PMC4150901; doi:10.1007/s11357-014-9673-9)
Supplement: Supplementary file 3 — (DOC 42 kb) [file 11357_2014_9673_MOESM3_ESM.doc]

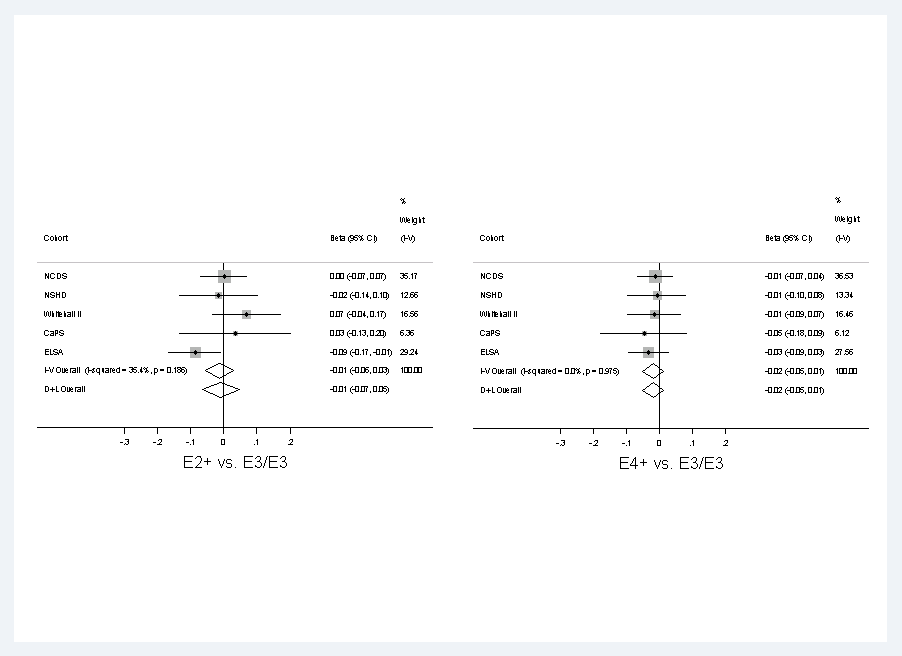


**Figure S3 Meta-analysis for the Association between *APOE* genotype and Semantic Fluency**

Adjusted for age and sex. ε2/ε4 participants were excluded from this analysis
