# Supplementary material for: Associations between APOE and low-density lipoprotein cholesterol genotypes and cognitive and physical capability: the HALCyon programme
Source: Age (Dordr). 2014 Jul 30;36(4):9673. doi: 10.1007/s11357-014-9673-9 (PMC4150901; doi:10.1007/s11357-014-9673-9)
Supplement: Supplementary file 6 — (DOC 44 kb) [file 11357_2014_9673_MOESM6_ESM.doc]

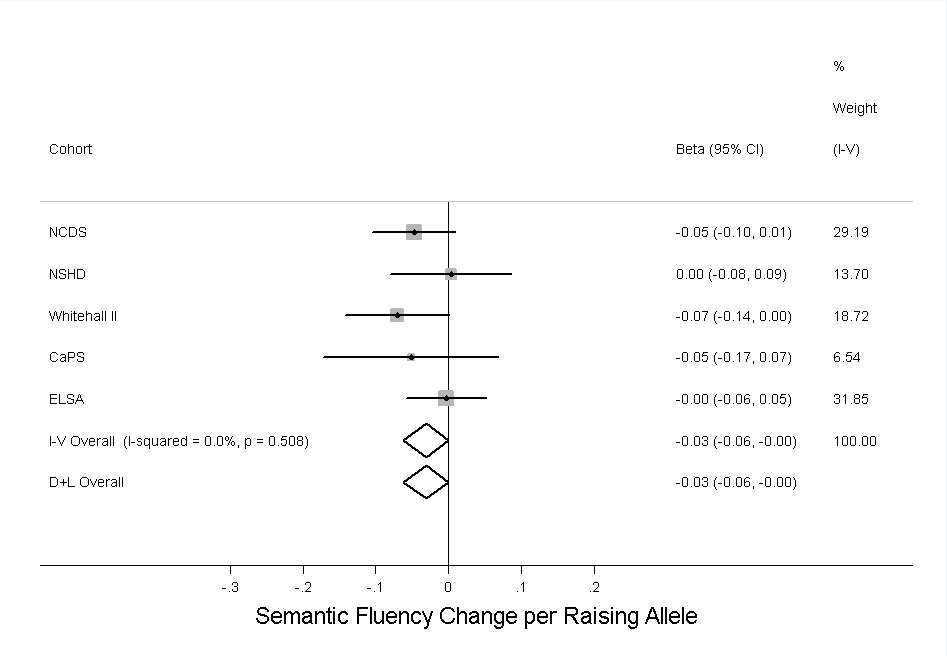


**Figure S6 Meta-analysis for the Association between rs2228671 *LDLR* and Semantic Fluency**

Adjusted for age and sex.
