# Supplementary material for: Associations between APOE and low-density lipoprotein cholesterol genotypes and cognitive and physical capability: the HALCyon programme
Source: Age (Dordr). 2014 Jul 30;36(4):9673. doi: 10.1007/s11357-014-9673-9 (PMC4150901; doi:10.1007/s11357-014-9673-9)
Supplement: Supplementary file 13 — (DOC 40 kb) [file 11357_2014_9673_MOESM13_ESM.doc]

Table S1 Summary of Physical Capability and Cognitive Capability by Cohort

|  |  |  |  | Cohort |  |  |  |  |
| --- | --- | --- | --- | --- | --- | --- | --- | --- |
| Measure | NCDS | NSHD | Whitehall II | CaPS | ELSA | HCS | Boyd Orr | LBC1921 |
| Word recall | 6.0 (1.5) | 24.0 (6.3) | 7.0 (2.4) | - | 5.0 (1.7) | - | - | - |
| Phonemic fluency | - | - | 16.0 (4.1) | - | - | - | - | 40.1 (12.3) |
| Semantic fluency | 22.5 (6.3) | 23.6 (6.9) | 16.0 (3.7) | 16.5 (4.8) | 20.2 (6.1) | - | - | - |
| Search Speed | 333.3 (86.9) | 282.4 (76.5) | - | - | 298.9 (90.4) | - | - | - |
| Grip strength, kg | - | 37.8 (14.3) | - | - | 32.0 (11.6) | 35.8 (11.0) | - | 26.5 (9.1) |
| Get up & go or walking speed, m/s | - | - | - | 0.60 (0.13) | 0.93 (0.30) | 0.57 (0.09) | 0.67 (0.16) | 1.40 (0.37) |
| Timed chair rises | - | 5.2 (1.7) | - | - | 9.6 (3.2) | 6.2 (1.5) | - | - |
| Ability to balance ≥5s, % | - | 96 | - | 63 | 87 | 82 | 61 | - |

Measures presented as mean (standard deviation), unless stated otherwise.

Word recall: 10 words in NCDS and ELSA; 20 in Whitehall II; 45 in NSHD. Phonemic fluency: 1 letter in Whitehall II; 3 letters in LBC1921. Search speed: 600 words in NSHD; 780 in NCDS and ELSA. Timed get up & go: CaPS, HCS, Boyd Orr. 2.44m walk: ELSA. 6m walk: LBC1921. Chair rises in reciprocal of time taken in seconds x 100: five rises in ELSA and HCS, ten in NSHD. Balance: Flamingo in NSHD, CaPS, HCS, Boyd Orr and Tandem in ELSA.
