# Supplementary material for: Associations between APOE and low-density lipoprotein cholesterol genotypes and cognitive and physical capability: the HALCyon programme
Source: Age (Dordr). 2014 Jul 30;36(4):9673. doi: 10.1007/s11357-014-9673-9 (PMC4150901; doi:10.1007/s11357-014-9673-9)
Supplement: Supplementary file 14 — (DOC 34 kb) [file 11357_2014_9673_MOESM14_ESM.doc]

**Table S2 Within-study Interactions between *APOE*** ε4 carrier status and Age in Cognitive Capability

| Measure | Cohort | Interaction Beta (95% CI) | p | N |
| --- | --- | --- | --- | --- |
| Word Recall | Whitehall II | -0.004 (-0.017- 0.009) | 0.55 | 3044 |
|  | ELSA | -0.006 (-0.012- 0.000) | 0.044 | 4971 |
| Phonemic Fluency | Whitehall II | -0.001 (-0.014- 0.012) | 0.84 | 3034 |
| Semantic Fluency | Whitehall II | 0.001 (-0.012- 0.014) | 0.86 | 3044 |
|  | CAPS | -0.001 (-0.030- 0.028) | 0.95 | 1190 |
|  | ELSA | 0.001 (-0.005- 0.007) | 0.74 | 4974 |
| Search Speed | ELSA | -0.006 (-0.012- 0.001) | 0.08 | 4901 |

Coefficients based on z-scores and adjusted for sex. Coefficients for *APOE* ε4+:carrier vs. non ε4 carrier
