# Supplementary material for: Associations between APOE and low-density lipoprotein cholesterol genotypes and cognitive and physical capability: the HALCyon programme
Source: Age (Dordr). 2014 Jul 30;36(4):9673. doi: 10.1007/s11357-014-9673-9 (PMC4150901; doi:10.1007/s11357-014-9673-9)
Supplement: Supplementary file 15 — (DOC 48 kb) [file 11357_2014_9673_MOESM15_ESM.doc]

**Table S3 Associations between *APOE* ε4 Carrier Status and Annual Change in Cognitive Capability**

| Measure | Cohort | Phases | Beta (95% CI) | p | N | I^2^; Het p |
| --- | --- | --- | --- | --- | --- | --- |
| Word Recall | NSHD | 2009†-1989 (~20y) | 0.051 (-0.057- 0.160) | 0.93 | 1651 |  |
|  | Whitehall II | Phase VII-Phase V (~5y) | -0.053 (-0.136- 0.030) | 0.21 | 2839 |  |
|  | ELSA | Phase III-Phase I (~4y) | -0.046 (-0.114- 0.023) | 0.19 | 4283 |  |
|  | Pooled |  | -0.027 (-0.83- 0.029) | 0.35 | 8773 | 24.9; 0.26 |
| Phonemic Fluency | Whitehall II | Phase VII-Phase V (~5y) | -0.060 (-0.144- 0.023) | 0.16 | 2828 |  |
|  | LBC 1921 | Wave II-Wave I (~4y) | 0.005 (-0.274- 0.285) | 0.97 | 292 |  |
|  | Pooled |  | -0.055 ( -0.135- 0.025) | 0.18 | 3120 | 0.0; 0.66 |
| Semantic Fluency | Whitehall II | Phase VII-Phase V (~5y) | -0.062 (-0.145- 0.022) | 0.15 | 2832 |  |
|  | CaPS | Phase V-Phase III (~12y) | -0.014 (-0.178- 0.150) | 0.87 | 757 |  |
|  | ELSA | Phase III-Phase I (~4y) | -0.023 (-0.092- 0.046) | 0.51 | 4281 |  |
|  | Pooled |  | -0.036 (-0.087- 0.014) | 0.16 | 7870 | 0.0; 0.75 |
| Search Speed | NSHD | 2009†-1989 (~20y) | 0.017 (-0.090- 0.123) | 0.76 | 1688 |  |
|  | ELSA | Phase III-Phase II (~2y) | 0.044 (-0.026- 0.114) | 0.22 | 4095 |  |
|  | Pooled |  | 0.036 (-0.023- 0.094) | 0.23 | 5783 | 0.0; 0.68 |

Coefficients based on z-scores for annual rate of change and adjusted for age and sex. Coefficients for *APOE* ε4+:carrier vs. non ε4 carrier.

†Data from NSHD collected between 2006 and 2011.
