# Supplementary material for: Associations between APOE and low-density lipoprotein cholesterol genotypes and cognitive and physical capability: the HALCyon programme
Source: Age (Dordr). 2014 Jul 30;36(4):9673. doi: 10.1007/s11357-014-9673-9 (PMC4150901; doi:10.1007/s11357-014-9673-9)
Supplement: Supplementary file 16 — (DOC 32 kb) [file 11357_2014_9673_MOESM16_ESM.doc]

**Table S4 Summary of Pooled Associations between Allelic Count of LDL-C-Related Genotypes** and Cognitive Capability

| Measure | Beta (95% CI) | p | I^2^ %; Het p | N |
| --- | --- | --- | --- | --- |
| Word recall | 0.00 (-0.02- 0.01) | 0.78 | 0.0; 0.76 | 15361 |
| Phonemic Fluency | 0.00 (-0.03- 0.03) | 0.98 | 0.0; 0.52 | 3521 |
| Semantic Fluency | -0.01 (-0.03- 0.01) | 0.27 | 52.1; 0.08 | 16529 |
| Search Speed | 0.00 (-0.02- 0.02) | 0.89 | 0.0; 0.68 | 12214 |

Coefficients based on z-scores and adjusted for age and sex. Allelic count of number of LDL-C-raising alleles for SNPs and number of ε4 alleles for *APOE*, for participants with all genotypes available.
