# Supplementary material for: Associations between APOE and low-density lipoprotein cholesterol genotypes and cognitive and physical capability: the HALCyon programme
Source: Age (Dordr). 2014 Jul 30;36(4):9673. doi: 10.1007/s11357-014-9673-9 (PMC4150901; doi:10.1007/s11357-014-9673-9)
Supplement: Supplementary file 17 — (DOC 39 kb) [file 11357_2014_9673_MOESM17_ESM.doc]

**Table S5 Within-study Interactions between *APOE*** ε4 carrier status and Age in Physical Capability

| Measure | Cohort | Interaction Beta (95% CI) | p | N |
| --- | --- | --- | --- | --- |
| Grip Strength | ELSA | 0.001 (-0.003- 0.005) | 0.77 | 4974 |
|  | HCS | 0.011 (-0.007- 0.029) | 0.24 | 2710 |
| TUG/Walk Speed | CAPS | 0.014 (-0.022- 0.050) | 0.45 | 748 |
|  | ELSA | 0.002 (-0.007- 0.011) | 0.68 | 3183 |
|  | HCS | 0.016 (-0.019- 0.051) | 0.36 | 2027 |
|  | Boyd Orr | -0.004 (-0.059- 0.050) | 0.88 | 361 |
| Timed Chair Rises | ELSA | 0.001 (-0.006- 0.008) | 0.72 | 4328 |
|  | HCS | 0.010 (-0.029- 0.049) | 0.63 | 1438 |
|  |  |  |  |  |
|  |  | OR (95% CI) | p | n/N |
| Ability to Balance ≥5s | CAPS | 1.029 (0.951- 1.115) | 0.48 | 477/757 |
|  | ELSA | 0.999 (0.977- 1.021) | 0.90 | 4379/5020 |
|  | HCS | 1.092 (0.982- 1.215) | 0.10 | 1227/1485 |
|  | Boyd Orr | 0.896 (0.789- 1.018) | 0.09 | 220/360 |

Coefficients for continuous outcomes based on z-scores. Adjusted for sex. Coefficients for *APOE* ε4+:carrier vs. non ε4 carrier
