# Supplementary material for: Associations between APOE and low-density lipoprotein cholesterol genotypes and cognitive and physical capability: the HALCyon programme
Source: Age (Dordr). 2014 Jul 30;36(4):9673. doi: 10.1007/s11357-014-9673-9 (PMC4150901; doi:10.1007/s11357-014-9673-9)
Supplement: Supplementary file 18 — (DOC 37 kb) [file 11357_2014_9673_MOESM18_ESM.doc]

**Table S6 Associations between *APOE* ε4 Carrier Status and Annual Change in Physical Capability**

| Measure | Cohort | Phases | Beta (95% CI) | p | N | I^2^; Het p |
| --- | --- | --- | --- | --- | --- | --- |
| Grip Strength | HCS | Phase II-Phase I (~4y) | 0.005 (-0.185- 0.194) | 0.96 | 583 |  |
|  | NSHD | 2009†-1999 (~10y) | -0.030 (-0.139- 0.078) | 0.58 | 1662 |  |
|  | LBC 1921 | Wave II-Wave I (~4y) | 0.116 (-0.158- 0.391) | 0.41 | 294 |  |
|  | Pooled |  | -0.007 (-0.096- 0.082) | 0.88 | 2539 | 0.0; 0.62 |
| Timed Walk Speed | ELSA | Phase III-Phase I (~4y) | -0.111 (-0.207- -0.016) | 0.02 | 2195 |  |
| Timed Chair Rises | NSHD | 2009†-1999 (~10y) | 0.078 (-0.029- 0.186) | 0.15 | 1652 |  |

Coefficients based on z-scores for annual rate of change and adjusted for age and sex. Coefficients for *APOE* ε4+:carrier vs. non ε4 carrier.

†Data from NSHD collected between 2006 and 2011.
