# Supplementary material for: Associations between APOE and low-density lipoprotein cholesterol genotypes and cognitive and physical capability: the HALCyon programme
Source: Age (Dordr). 2014 Jul 30;36(4):9673. doi: 10.1007/s11357-014-9673-9 (PMC4150901; doi:10.1007/s11357-014-9673-9)
Supplement: Supplementary file 19 — (DOC 34 kb) [file 11357_2014_9673_MOESM19_ESM.doc]

**Table S7 Summary of Pooled Associations between Allelic Count of LDL-C-Related Genotypes and Physical** Capability

| Measure | Beta (95% CI) | p | I^2^ %; Het p | N |
| --- | --- | --- | --- | --- |
| Grip Strength | 0.00 (-0.01- 0.02) | 0.66 | 36.2; 0.20 | 10230 |
| TUG/Walk Speed | 0.01 (-0.02- 0.05) | 0.44 | 42.8; 0.14 | 6579 |
| Timed Chair Rises | -0.03 (-0.05- -0.01) | 0.01 | 0.0; 0.72 | 7821 |
|  |  |  |  |  |
|  | OR (95% CI) | p | I^2^ %; Het p | n/N |
| Ability to Balance ≥5s | 1.00 (0.94- 1.06) | 0.98 | 3.4; 0.39 | 8309/9652 |

Coefficients for continuous outcomes based on z-scores and adjusted for age and sex. Allelic count of number of LDL-C-raising alleles for SNPs and number of ε4 alleles for *APOE*, for participants with all genotypes available.
